# Improving Outcomes of Iatrogenic Type A Aortic Dissection during Cardiac Surgery

**DOI:** 10.1055/s-0039-1695729

**Published:** 2019-11-26

**Authors:** Nicholas J. Shea, Antonio R. Polanco, Alex D'Angelo, Casidhe-Nicole Bethancourt, Joseph Sanchez, Isaac George, Virendra Patel, Hiroo Takayama

**Affiliations:** 1 Division of Cardiac, Thoracic, and Vascular Surgery, NYP/CU Medical Center, New York-Presbyterian/Columbia University Aortic Center (VP and HT), New York, New York

**Keywords:** iatrogenic aortic dissection, Type A aortic dissection, aortic surgery

## Abstract

**Background** Iatrogenic Type A aortic dissection (IAD) is a rare but devastating complication of cardiac and aortic surgery with reported operative mortality of 30 to 50%. In this study, we report our experience with IAD and propose a standardized approach to management.

**Methods** From January 1, 2000 through December 31, 2016, 23,275 patients underwent cardiac surgery at our institution. We identified 15 patients who developed IAD. Our approach to management included (1) immediate repair, (2) involvement of a second attending surgeon, (3) aggressive monitoring of malperfusion, (4) securing true lumen arterial perfusion access and systemic cooling, and (5) performance of hemiarch or total arch replacement based on the presence of suspected brain malperfusion. The index operation was also completed at the same time. Patient preoperative characteristics, operative sequence and technique, complications, and outcomes were analyzed with chart review.

**Results** The incidence of IAD at our institution was 0.06% ( *n* = 15). A disproportionate percentage of patients had aneurysmal ascending aortas (33.3%). The index surgery consisted of aortic surgery in five patients (33.3%), coronary bypass in three patients, valve surgery in five patients, and transplantation in one patient. The mechanism of dissection was aortic cannulation in 66.7% and aortic root vent site cannulation in 13.3%. In 46.7% of patients, the IAD was first recognized based on clinical evidence such as aortic hematoma, pericardial bleeding, or abnormal perfusion line pressures. In 40.0%, the diagnosis was made with intraoperative echocardiography without any clinical manifestations. The timing of the diagnosis was at the initiation of cardiopulmonary bypass initiation in 60.0%, while in 40.0% it was recognized after discontinuation of bypass. Hemiarch was done in 73.3% and total arch replacement performed in 13.3%. Isolated ascending repairs were done in two patients. Bypass and cross-clamp times were 229.5 ± 212.7 minutes and 130.5 ± 109.5 minutes, respectively. In-hospital mortality in our cohort was 6.7%. While stroke occurred in one patient, no visceral organ malperfusion was recognized.

**Conclusions** Incidence of IAD is low with cannulation of an aneurysmal aorta being a risk factor. A standardized approach may result in reduced operative mortality.

## Introduction

Iatrogenic Type A aortic dissection (IAD) is a rare and potentially fatal complication of cardiac or aortic surgery. 1 2 3 Although the operating room arguably is the best place to develop an acute Type A aortic dissection, operative mortality in IAD remains high at 25 to 48%. 1 2 3 4 5 Mortality in spontaneous Type A aortic dissection, by comparison, is around 15 to 30%. 6 7 8 9 10 Why reported mortality is so high in this entity and exceeds that of spontaneous acute Type A dissection remains an open question. Existing knowledge is limited in part by the rarity of this entity, and while outcomes from large databases have been reported, these data do not provide the granular clinical data necessary to develop a “best practice” approach to optimize outcomes of this devastating complication.

In the present study, we hypothesized that the outcomes of IAD treated in the contemporary era are better than earlier reports suggest. We studied patients with IAD at our institution with the specific objectives of examining preoperative characteristics, describing operative details, and measuring surgical outcomes in these patients. Based on these observations, we propose a set of general principles for management.

## Materials and Methods

### Data Collection

This is a retrospective study of 23,275 patients who underwent open cardiac or aortic surgical procedures at our institution between January 1, 2000 and December 31, 2016. Of these, we identified 15 patients who developed IAD during their primary indicated surgery. All but two of these cases underwent immediate surgical aortic repair. The identified incidence for IAD during this time period was 0.06%. The research protocol was approved by the Columbia University Medical Center Institutional Review Board and is compliant with the Health Insurance Portability and Accountability Act regulations. Individual consent was waived due to the retrospective nature of the study in accordance with our center's Institutional Review Board protocol. Initial data such as patient demographics, risk factors, operative information, and in-hospital outcomes were prospectively added to a New York-Presbyterian/Columbia University Aortic Center surgery database.

### Statistical Analysis

Data were obtained from chart review. Statistical procedures were done using Microsoft Excel (Microsoft; Redmond, Washington). Data are expressed as absolute values or mean ± standard deviation.

## Results

### Patient Characteristics

**Fig. 1:**
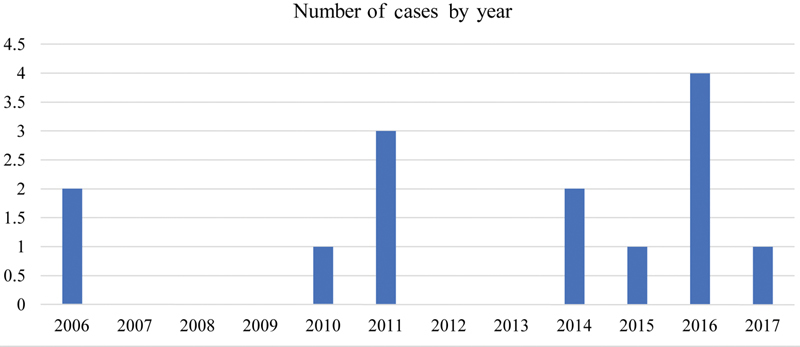
Number of iatrogenic aortic dissection cases by year.

Patient characteristics are presented in Table 1 . Sixty percent were women and mean age was 74 ± 6.8 years old. Notably, 33.3% of patients were undergoing procedures for aneurysmal disease at the time of the dissection, with one of 15 patients having an ascending aorta diameter of 4 to 5 cm and four having an ascending aorta diameter > 5 cm. History of hypertension was the most common comorbidity, present in nearly 90% of the cohort. A positive smoking history was seen in about a third. None of the identified patients had any known connective tissue disorder such as Marfan syndrome predisposing them to aortic dissection. The distribution by year are shown in Fig. 1 .

**Table 1:** Patient characteristics

Characteristics	*n*	%
Female	9	60.0
Age	74 ± 7.3	
*Aortic size* :		
Normal ascending/root	10	66.7
Ascending 4–5cm	1	6.7
Ascending > 5cm	4	26.7
Previous aortic intervention	2	13.3
Family history of aortic disease	1	6.7
Hypertension	13	86.7
Hyperlipidemia	9	60.0
Positive smoking history	4	26.7
Coronary artery disease	5	33.3
Diabetes, Type II	2	13.3
Atrial fibrillation	5	33.3
Congestive heart failure	4	26.7
Aortic stenosis	5	33.3
Chronic lung disease	4	26.7
Average preoperative ejection fraction	58.3% ± 5.0%	
*Index operation* :		
Ascending aneurysm repair with root repair	3	20.0
Ascending aneurysm repair without root repair	2	13.3
Isolated valve replacement	4	26.7
Isolated coronary artery bypass grafting	3	20.0
CABG + valve replacement	2	13.3
Heart transplant	1	6.7

Abbreviations: CABG, coronary artery bypass grafting.

### Intraoperative Course

Mechanism, timing of diagnosis, presentation, and extent of dissection data are presented in Table 2 . Most (66.7%) dissections occurred during aortic cannulation. The second most common mechanism of dissection was aortic clamping. For the majority of patients, dissection was immediately evident at the time of cannulation, with critical intraoperative signs of dissection being bleeding from the aorta into the pericardium, bleeding at the cannula site, abnormal aortic perfusion line pressures, hemodynamic collapse or clinical tamponade, an expanding aortic hematoma, and failure to achieve cardioplegic arrest. These clinical manifestations prompted detailed examination with transesophageal echocardiography (TEE) and/or epiaortic ultrasound, which confirmed the diagnosis. Of note, however, in 6 of 15 patients (40.0%), the initial diagnosis of IAD was made by the anesthesiologist with TEE rather than by direct observation of the surgical field. In 13.3% of cases, the diagnosis was made by TEE after decannulation. In one patient, the IAD was discovered shortly after arrival to the intensive care unit (ICU), presenting with hypotension. With regard to the anatomic extent of the dissection, most frequently they were limited to zone 0. In two patients, however, the dissection extended distal to zone 4. Only one of these three patients had an aneurysm of the descending aorta.

**Table 2:** Iatrogenic aortic dissection details

Variable	*N*	%
*Mechanism/timing of injury* :		
Aortic cannulation	10	66.7
Aortic root vent site	2	13.3
Aortic cross-clamp or partial-occluding clamp	1	6.7
Innominate artery cannulation	1	6.7
Proximal anastomotic site of vein graft	1	6.7
*Timing of diagnosis* :		
Immediately following cannulation	9	60.0
Following decannulation	2	13.3
At time of chest closure	1	6.7
During cardioplegia administration	1	6.7
Immediately following clamp removal	1	6.7
After ICU admission	1	6.7
*Presentation* :		
Discovered on TEE	6	40.0
Bleeding from aorta into pericardium	3	20.0
Bleeding from cannula site	2	13.3
Expanding hematoma	1	6.7
Abnormal aortic perfusion line pressures	1	6.7
No arrest with cardioplegia	1	6.7
Hypotension in ICU, emergent open chest	1	6.7
*Extent of dissection* :		
Isolated zone 0	9	60.0
Including zone 0, zone 1, and zone 2	4	26.7
Including zone 3 to distal zone 4	1	6.7
Zone 0 through distal to zone 4	1	6.7

Abbreviations: ICU, intensive care unit; TEE, transesophageal echocardiography.

**Fig. 2:**
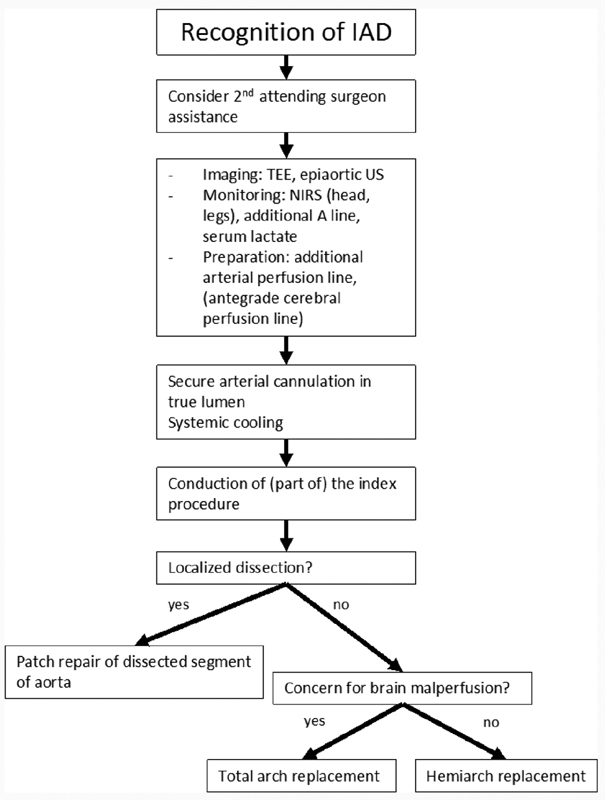
Approach to the management of iatrogenic Type A aortic dissection (IAD). NIRS, near infrared spectroscopy; TEE, transesophageal echocardiography; US, ultrasound.

In general, we applied the management protocol depicted in Fig. 2 . At our institution, a second attending surgeon is usually involved to provide further assistance. (Our standard for routine cardiac cases is one attending surgeon for a case.) In 2 of 15 cases, a vascular surgery attending was already present as part of the planned procedure for the management of the pre-existing descending aortic disease; in one case, a second cardiothoracic attending was already present as part of the planned procedure to deploy a thoracic endovascular aortic repair graft; and in four cases, a second cardiothoracic surgery attending was called in to assist.

Once the diagnosis was confirmed, arterial perfusion of the true lumen was immediately established, and systemic cooling followed. The cannulation site was changed to obtain true lumen perfusion access in 7 of 15 patients (46.7%). Average core temperature was 22.9 ± 4.2°C. Meanwhile, malperfusion was carefully monitored: epiaortic ultrasound was performed to examine the flow in the innominate, left carotid, and left subclavian arteries; TEE was performed to assess the flow in the true and false lumens in the descending aorta; near infrared spectroscopy (NIRS) was applied in the bilateral forehead and in the lower extremities; an additional arterial pressure monitoring line was placed; and serum lactate and urine output were closely followed.

When the IAD was recognized before completion of the index operation, as in the majority of patients, the index operation was completed at the same time. As noted earlier, specific procedural technique and sequence were at the discretion of the attending surgeon. We frequently perform part or all of the index operation while the patient is being cooled to minimize cardiopulmonary bypass time.

Table 3 describes the aortic repair strategy and operative details. Hemiarch replacement was performed in most patients ( *n* = 11, 73.3%). Total arch was done in two patients (13.3%). Isolated ascending aorta replacement was performed in two patients (13.3%). In one of these patients, the dissection extended into the arch but the arch was severely calcified precluding hemiarch or arch replacement. In the other patient, the tear was a full-thickness tear of the aortic cannulation site without an extensive dissection plane, allowing a more limited patch repair. Both of these patients survived their hospital stay.

**Table 3:** Operative details

Variable	*n*	*%*
*Repair strategy for the IAD* :		
Root repair a	4	26.7
Bioroot	3	20.0
VSARR	1	6.7
Ascending aortic replacement	2	13.3
Hemiarch replacement	11	73.3
Total arch replacement	2	13.3
Cross-clamp time (min)	130.5 ± 109.5	
Cardiopulmonary bypass time (min)	229.5 ± 212.7	
Deep hypothermic arrest time (min)	12.8 ± 7.1	
Selective antegrade cerebral perfusion (min)	14.3 ± 15.6	
Core temperature (deg C)	22.9 ± 4.2	

Abbreviations: IAD, iatrogenic Type A aortic dissection; VSARR, valve sparing aortic root replacement.

a Two bioroots and 1 VSARR were the index procedure, while 1 bioroot was required for root dissection.

Root repair was performed in four (26.7%) patients, three of whom had preoperative aortic root enlargement and replacement of the root was planned. In only one patient, the root was replaced because the dissection extended to the level of the coronaries. Of these four patients, in three patients the root was replaced with a tissue valve-conduit (“Bio-Bentall”), and in one patient a valve sparing root replacement (“David V”) was performed.

Cross-clamp time averaged 130.5 ± 109.5 minutes (range: 92–935 minutes). Average cardiopulmonary bypass time was 229.5 ± 212.7 minutes (range: 0–455 minutes).

### Outcomes

There was one patient with in-hospital death with in-hospital mortality of 6.7%. This patient died of sepsis and multisystem organ failure. Postoperative morbidity outcomes are shown in Table 4 . Renal failure, as defined by the Society of Thoracic Surgeons database, was seen in four patients (26.7%). One patient (6.7%) experienced stroke, which was confirmed with imaging and diagnosed by an attending neurologist. This patient did not have evidence of intraoperative malperfusion but suffered several small strokes with left hemiplegia due to cardiogenic emboli. Five patients suffered pneumonias (33.3%) and three patients underwent tracheostomy for postoperative respiratory failure (20.0%). Four patients required return to the operating room for washout and chest exploration (26.7%) and one patient required venoarterial extracorporeal membrane oxygenation for cardiogenic shock (6.7%). This patient did well and was alive at last known follow-up 825 days post surgery.

**Table 4:** Morbidity and mortality

	*n*	%
In-hospital mortality	1	6.7
Postoperative renal failure	4	26.7
Stroke	1	6.7
Pneumonia	5	33.3
Tracheostomy	3	20.0
Prolonged ventilation time (≧ 21days)	3	20.0
Return to OR/washout	4	26.7
ECMO	1	6.7
Average ICU stay (days)	12.7 ± 14.0	
Average mechanical ventilator time (days)	7.9 ± 14.4	
Time to discharge from surgery (days)	20.7 ± 15.7	

Abbreviations: ECMO, extracorporeal membrane oxygenation; ICU, intensive care unit; OR, operating room.

The average ICU stay was 12.7 ± 14.0 days. Time to discharge from the date of initial surgery was 20.7 ± 15.7 days.

## Discussion

The present study adds to the literature in several important ways, including confirming the low incidence (0.06%) of this iatrogenic complication, providing a more detailed clinical picture of this entity (e.g., patient characteristics, mechanism, manifestation, and diagnosis) and offering evidence of better operative mortality (6.7%).

According to published reports, the mortality from IAD has persistently been high, while it occurs only in 0.06 to 0.23% of cardiac surgical procedures. 1 2 3 The largest of these single center series, published by Leontyev et al, includes 48 patients, of which 31 patients were undergoing cardiac surgery and 12 were undergoing cardiac catheterization at the time of dissection. 4 In that series, the authors report 41.7% 30-day mortality with significant predictors of death being preoperative New York Heart Association (NYHA) class IV functional status and coronary malperfusion. 4 Still et al published one of the earlier series of IAD. 1 In their cohort of 24 patients, mortality was 25%, with ventricular dysfunction from myocardial ischemia being the primary cause of death. Fleck et al report 43% operative and 43% overall mortality at a median follow-up of 20 months with decompensated heart failure and multisystem organ failure being the primary causes of death. 2 Narayan et al analyzed a large database including over 15,000 patients and found that the IAD incidence was 0.04% and mortality was 33%. 5 That study identified older age and history of atheromatous disease as risk factors for IAD. 5 Using the Society of Thoracic Surgeons database, Williams et al analyzed 2.2 million cardiac procedures from 1996 to 2007 and found a 0.06% incidence of IAD with 48% operative mortality. 3

In this report, we found similar incidence of IAD to that reported in the literature; however, our operative mortality was relatively low at 6.7% (1/15). Most dissections were related to aortic cannulation and they were most frequently diagnosed at the time of dissection by the surgeon based on clinical evidence or observations made in the surgical field. A greater proportion of the patients who developed IAD at our institution had aneurysms when compared with our general cardiac surgical population, offering some evidence to support the conventional wisdom that patients with aortic aneurysms are at greater risk of IAD than the typical cardiac surgical patient.

Notably, many iatrogenic Type A dissections at our institution were limited ascending tears confined to zone 0 of the aorta, which may be one reason for the relatively low mortality among our patients. Nearly all of our patients who suffered IAD also had normal ejection fraction, which may contribute to our improved survival. As noted above, several studies have identified ventricular dysfunction and higher NYHA class as risk factors for mortality following IAD.

In the context of these patient characteristics, our approach to management, summarized in Fig. 1 and described below, may offer patients with IAD the best opportunity at survival. Specifically, we provide intraoperative monitoring during cardiac and aortic procedures with TEE, pulmonary artery pressure monitoring with a Swan-Ganz catheter, and bispectral index (or NIRS for aortic surgery). IAD is managed with the following principles: (1) immediate repair, (2) involvement of a second attending surgeon, (3) aggressive monitoring for malperfusion, (4) securing true lumen arterial perfusion access and systemic cooling, and (5) performance of hemiarch or total arch replacement based on the presence of suspected brain malperfusion with completion of the index procedure. The detail of procedural technique is left to the discretion of the attending surgeon.

The inferences that can be drawn from the results in this study are limited by the relatively small number of patients included, an inevitable feature of this complication's rarity; intrinsic heterogeneity between cases resulting from patient characteristics, surgeon preferences, and operative techniques; and limited post-operative follow-up. Like many current reports on IAD, this study is a retrospective, descriptive account of one institution's experience.

## Conclusion

Overall, the best approach to this complication, as has been noted by others, is avoidance. Our experience along with that of other centers makes clear, however, that it can occur in all manner of operations and in all types of patients. The extent and timing of the dissection can vary widely as well, and the approach to repair and operative sequence therefore should be appropriately tailored to the patient and the clinical scenario. Nevertheless, applying certain principles may result in reduced operative and overall mortality.

## Notes

**Editor's Questions:**

1. Please describe how you reposition your cannula to achieve true lumen perfusion?

True lumen cannulation for systemic perfusion on cardiopulmonary bypass is critical to successful management of iatrogenic Type A dissection. At the time of dissection, we assess whether there is true lumen access using epiaortic ultrasound, transesophageal echo, and tests of line pressures. When cannulation of the true lumen cannot be confirmed, there appears to be a sufficient nondissected area of the ascending aorta; the cannula site may be changed to this area of the ascending aorta safely using Seldinger technique with introduction of the aortic cannula over a wire under transesophageal echocardiography and epiaortic ultrasound guidance. Otherwise, we obtain true lumen perfusion with cannulation of the femoral or axillary arteries. In our cohort, the cannulation site was changed in seven patients. In three patients, it was changed to the femoral artery; in three patients; it was changed to the axillary artery; and in one patient, it was changed to a nondissected part of the ascending aorta using Seldinger technique as described.
